# Association of coexisting celiac disease with macrovascular and microvascular complications in adults with type 2 diabetes mellitus: a retrospective propensity score–matched cohort study

**DOI:** 10.1007/s12020-026-04769-3

**Published:** 2026-09-28

**Authors:** Renan Prado, Melissa Lou Silva

**Affiliations:** 1 Department of Internal Medicine, Cleveland Clinic Main Campus, Cleveland, OH 44195 USA; 2 Liver Imaging Group, Department of Radiology, University of California San Diego, La Jolla, 92122 CA USA

**Keywords:** Diabetes complications, Celiac disease, Type 2 diabetes mellitus, Gluten free diet, Hyperglycemia

## Abstract

**Background:**

Celiac disease (CD) and type 2 diabetes mellitus (T2DM) are increasingly recognized as overlapping metabolic conditions. However, the impact of concomitant CD on diabetes-related complications and metabolic outcomes in patients with T2DM remains poorly characterized. We aimed to compare clinical outcomes between patients with T2DM and CD versus patients without CD.

**Methods:**

We performed a retrospective multicenter electronic health record (EHR)-based cohort study using the TriNetX research network. Adults with T2DM and concomitant CD were identified using ICD-10-CM codes for T2DM and CD, along with either markedly elevated tissue transglutaminase IgA levels or upper endoscopy procedures.

Propensity score matching (1:1) was performed for demographics, cardiovascular risk factors, lipid disorders, medications, and laboratory markers, yielding 8,760 patients per cohort.

Outcomes included albuminuria, depression, anxiety, severe hyperglycemia, ophthalmic complications, hospitalization, neuropathy, nephropathy, and a composite cardiovascular outcome.

**Results:**

After matching, patients with T2DM and CD had significantly higher hazards of albuminuria (HR 1.30, 95% CI 1.15–1.46), depression (HR 1.14, 95% CI 1.07–1.21), anxiety (HR 1.12, 95% CI 1.06–1.18), severe hyperglycemia (HR 1.39, 95% CI 1.27–1.53), ophthalmic complications (HR 1.77, 95% CI 1.55–2.02), neuropathy (HR 1.73, 95% CI 1.59–1.88), nephropathy (HR 1.13, 95% CI 1.04–1.22), and a composite cardiovascular outcome (HR 1.09, 95% CI 1.01–1.17) compared with matched controls. Hospitalization risk did not differ between cohorts (HR 0.97, 95% CI 0.92–1.01). Mean follow-up after matching were 1,574 days and 1,438 days.

**Conclusions:**

Among patients with T2DM, concomitant CD was associated with significantly higher risks of macro and microvascular diabetic complications, and psychiatric comorbidities.

**Supplementary Information:**

The online version contains supplementary material available at https://doi.org/10.1007/s12020-026-04769-3.

## Introduction

Celiac disease (CD) is a chronic, immune-mediated enteropathy triggered by dietary gluten ingestion in genetically predisposed individuals, with a global prevalence of approximately 0.5% to 2% [1, 2]. The disease is characterized by small intestinal villous atrophy, chronic inflammation, and nutrient malabsorption, and is increasingly recognized as a systemic autoimmune disorder with multiple extraintestinal manifestations [1, 3]. Up to 22% of patients with CD have neurologic or psychiatric manifestations, attributable to a combination of chronic inflammation, nutrient deficiencies, and adaptive immune responses extending beyond the intestinal mucosa [4]. 

While the association between CD and type 1 diabetes mellitus is well established with prevalence up to 16% in T1DM populations, the relationship between CD and type 2 diabetes mellitus (T2DM) remains less well characterized [5, 6]. Population-based studies have yielded conflicting results: a large Swedish cohort and Mendelian randomization analysis found no increased risk of T2DM among individuals with CD, while a case-control study demonstrated that patients with CD have a significantly lower prevalence of non-insulin-dependent diabetes and metabolic syndrome compared with matched controls [6, 7]. Conversely, the prevalence of T2DM among celiac patients appears comparable to that of the general population [8]. 

In patients with T1DM, concomitant CD has been identified as an independent risk factor for microvascular complications. A case-control study found that adults with T1DM and newly diagnosed CD had significantly higher prevalences of ophthalmologic complications (58.3% vs. 25%), nephropathy (41.6% vs. 4.2%), and worse glycemic control compared with those without CD [9]. 

However, whether concomitant CD similarly modifies the risk of diabetes-related complications in patients with T2DM has not been systematically investigated. Given the rising global burden of T2DM and the increasing recognition of CD as a systemic disease, understanding the clinical implications of this overlap is of considerable importance. We therefore aimed to compare clinical outcomes between patients with T2DM and concomitant CD versus those with T2DM alone, using a large, multicenter, retrospective propensity score–matched cohort from a national database.

## Methods

### Study design and data source

We performed a retrospective propensity score–matched cohort study using the TriNetX Research USA Minimal Date Shift network, which includes electronic health record data from 56 healthcare organizations across the United States [10]. Adult patients with type 2 diabetes mellitus (T2DM) were identified using ICD-10-CM code E11.

### Study population

The exposure cohort consisted of patients with concomitant celiac disease (CD), defined by ICD-10-CM code K90.0 in addition to either markedly elevated tissue transglutaminase IgA antibody levels (≥ 200 U/L) or a documented upper endoscopy/esophageal endoscopy procedure, to increase diagnostic specificity for clinically significant or biopsy-supported CD.

A markedly elevated tTG-IgA has a high positive predictive value for CD, while upper endoscopy represents the standard diagnostic pathway through which duodenal biopsy is typically obtained in routine clinical practice [11]. Although this approach does not confirm biopsy-proven CD in every patient, it was intended to preferentially identify individuals with clinically established CD and reduce potential diagnostic misclassification. Because this algorithm still permits inclusion of some patients with an ICD-10 code for CD but incomplete or seronegative diagnostic workup, any resulting misclassification would most likely dilute the distinction between exposed and unexposed groups, biasing observed associations toward the null rather than inflating them. The comparator cohort included patients with T2DM without celiac disease or type 1 diabetes mellitus (Supplementary Table 1).

The index date was defined as the first occurrence of the qualifying cohort criteria. Outcomes were assessed beginning 30 days after index.

### Covariates and propensity score matching

Propensity score matching was performed in a 1:1 fashion using nearest-neighbor matching without replacement. Matching variables included age, sex, race, ethnicity, hypertension, dyslipidemia, insulin use, metformin use, antilipidemic therapy, hemoglobin A1c (HbA1c), LDL cholesterol, and body mass index (BMI) (Supplementary Table 2). Propensity score matching was performed within the TriNetX platform using its built-in 1:1 nearest-neighbor matching algorithm. The platform automatically applies its predefined matching parameters, including the matching distance, which are not user-configurable.

Important factors including duration of diabetes, duration of celiac disease, gluten-free diet adherence, socioeconomic status, diabetes severity, medication adherence, and healthcare utilization are incompletely captured within TriNetX and may contribute to residual confounding.

### Outcomes

The primary outcomes were incident diabetic microvascular and metabolic complications identified using ICD-10-CM diagnosis codes, including albuminuria/proteinuria (ICD-10: R80), diabetic neuropathy (ICD-10: E11.4), diabetic nephropathy (ICD-10: E11.2), diabetic ophthalmic complications (ICD-10: E11.3), and severe hyperglycemia. Severe hyperglycemia is defined as at least one value of HbA1c ≥ 10%.

Secondary outcomes included depression (ICD-10: F32), anxiety (ICD-10: F41), hospitalization, and a composite cardiovascular outcome. This outcome was defined as the first occurrence of any of the following, identified using ICD-10-CM diagnosis codes: acute myocardial infarction (I21), subsequent ST-elevation and non-ST-elevation myocardial infarction (I22), cardiac arrest (I46), heart failure (I50), nontraumatic intracerebral hemorrhage (I61), other nontraumatic intracranial hemorrhage (I62), cerebral infarction (I63), occlusion and stenosis of precerebral arteries without infarction (I65), occlusion and stenosis of cerebral arteries without infarction (I66), ventricular fibrillation and flutter (I49.0), ventricular premature depolarization (I49.3), other specified cerebrovascular disease (I67.8), and unspecified cerebrovascular disease (I67.9). Because this composite is broader than the components conventionally used to define major adverse cardiovascular events (MACE) — typically limited to myocardial infarction, stroke, and cardiovascular death — to also include heart failure, cardiac arrest, and high-risk arrhythmias, we refer to it as a composite cardiovascular outcome rather than MACE to avoid terminological ambiguity. Substantial heterogeneity in the composition of MACE definitions has previously been documented: a Cochrane review of biomarkers for preoperative cardiac risk prediction identified 80 different definitions of MACE across included studies [12], and a systematic review using administrative data found that only 8.6% of studies used the traditional three-point definition, with wide variation in both the components and the diagnostic codes used to define them [13]. Similarly broad composite cardiovascular outcome definitions, including arrhythmia-related ICD-10 codes, have been used in other large database studies [14]. 

Hospitalization was defined as any all-cause inpatient encounter occurring after the index washout period. For the time-to-event analysis, only the first hospitalization per patient was considered; therefore, the hazard ratio reflects the relative hazard of experiencing at least one hospitalization rather than differences in the total number of hospitalizations between cohorts. Repeat hospitalizations within the same patient were not counted as additional events in this analysis. Incident events were defined as the first occurrence of the relevant diagnosis code following the 30-day post-index washout period among patients without a prior diagnosis of that outcome.

Severe hyperglycemia was an exception to this approach: because it was defined by a laboratory threshold (HbA1c ≥ 10%) rather than a diagnosis code, patients with a pre-index HbA1c ≥ 10% were not excluded from being at risk for this outcome. For this outcome, we report two complementary analyses: a general definition, capturing any qualifying HbA1c ≥ 10% value occurring after index during follow-up regardless of prior HbA1c status, and a first post-index occurrence, identifying the first qualifying HbA1c ≥ 10% value occurring after the index date, excluding patients with prior HbA1c ≥ 10% before index event. These two approaches therefore differ in their relationship to incident disease. The general definition reflects the occurrence of a qualifying HbA1c value at any point during follow-up, including among patients with pre-index elevation, and should not be interpreted as a strictly incident event in the diagnosis-code sense used for the other outcomes. The first post-index occurrence, by excluding patients with a pre-index HbA1c ≥ 10%, more closely approximates a strictly incident event and is more directly comparable to the incident diagnosis-code–based outcomes reported elsewhere.

The complete list of ICD-10-CM codes used to define study outcomes is provided in Supplementary Table 3.

### Statistical analysis

Time-to-event analyses were performed using Kaplan–Meier methodology with hazard ratios (HRs) calculated by Cox proportional hazards models. Statistical significance was defined as a two-sided p-value < 0.05. Covariate balance after matching was evaluated using standardized mean differences (SMDs), with values < 0.10 considered indicative of adequate balance.

## Results

Before matching, the cohort with concomitant T2DM and CD included 8,770 patients, while the comparator cohort with T2DM without CD included 6,294,516 patients. After propensity score matching, 8,760 patients remained in each cohort with improved balance across demographic, metabolic, and treatment-related variables. Although propensity score matching substantially improved baseline comparability, several variables remained modestly imbalanced, including age (SMD 0.105), hypertension (SMD 0.111), HbA1c (SMD 0.155) and LDL cholesterol (SMD 0.188). BMI showed a more substantial residual imbalance (SMD 0.472), exceeding the pre-matching SMD for this variable (0.337) and representing the largest residual imbalance among all matched covariates. Even after matching, patients with coexisting CD were younger (56.1 vs. 58.2 years), had a lower prevalence of hypertension (58.5% vs. 63.9%), lower mean HbA1c (7.0% vs. 6.7%), lower LDL cholesterol (91.8 vs. 99.1 mg/dL), and substantially lower BMI (30.3 vs. 34.5 kg/m²) than matched controls. Accordingly, the observed associations should be interpreted in the context of these remaining baseline differences.

Mean age after matching was 56.1 ± 21.4 years in the T2DM + CD cohort and 58.2 ± 18.4 years in the T2DM-only cohort. Mean follow-up duration after matching was 1,574 days in the T2DM + CD cohort and 1,438 days in controls (Table 1).

**Table 1:** Baseline characteristics before and after propensity score matching

Characteristic	T2DM + CD Before Matching (*n* = 8,770)	T2DM Without CD Before Matching (*n* = 6,294,516)	SMD Before	T2DM + CD After Matching (*n* = 8,760)	T2DM Without CD After Matching (*n* = 8,760)	SMD After
Age, mean ± SD (years)	56.1 ± 21.4	67.9 ± 14.9	0.642	56.1 ± 21.4	58.2 ± 18.4	0.105
Male sex, n (%)	3,007 (34.3%)	3,082,301 (49.9%)	0.319	3,007 (34.3%)	2,895 (33.0%)	0.027
White race, n (%)	7,078 (80.8%)	3,760,641 (60.9%)	0.449	7,076 (80.8%)	7,091 (80.9%)	0.004
Black race, n (%)	698 (8.0%)	1,237,010 (20.0%)	0.353	698 (8.0%)	717 (8.2%)	0.008
Asian race, n (%)	156 (1.8%)	306,136 (5.0%)	0.177	156 (1.8%)	273 (3.1%)	0.086
Hispanic ethnicity, n (%)	595 (6.8%)	564,996 (9.1%)	0.087	595 (6.8%)	571 (6.5%)	0.011
Non-Hispanic ethnicity, n (%)	6,827 (77.9%)	4,192,923 (67.9%)	0.228	6,825 (77.9%)	6,833 (78.0%)	0.002
Unknown ethnicity, n (%)	1,340 (15.3%)	1,420,581 (23.0%)	0.197	1,340 (15.3%)	1,356 (15.5%)	0.005
Hypertension, n (%)	5,127 (58.5%)	1,519,490 (24.6%)	0.733	5,127 (58.5%)	5,601 (63.9%)	0.111
Dyslipidemia, n (%)	4,991 (57.0%)	1,180,142 (19.1%)	0.847	4,989 (57.0%)	5,395 (61.6%)	0.094
Antilipemic agent use, n (%)	3,977 (45.4%)	1,206,991 (19.5%)	0.574	3,977 (45.4%)	4,315 (49.3%)	0.077
Insulin use, n (%)	4,250 (48.5%)	489,328 (7.9%)	1.010	4,248 (48.5%)	4,272 (48.8%)	0.005
Metformin use, n (%)	2,498 (28.5%)	687,003 (11.1%)	0.447	2,498 (28.5%)	2,825 (32.2%)	0.081
Hemoglobin A1c, mean ± SD (%)	7.0 ± 1.9	6.9 ± 1.7	0.074	7.0 ± 1.9	6.7 ± 1.8	0.155
LDL cholesterol, mean ± SD (mg/dL)	91.8 ± 37.5	99.9 ± 38.7	0.212	91.8 ± 37.5	99.1 ± 40.6	0.188
BMI, mean ± SD (kg/m²)	30.3 ± 8.8	33.2 ± 8.2	0.337	30.3 ± 8.8	34.5 ± 9.2	0.472

Abbreviations: BMI, body mass index; CD, celiac disease; LDL, low-density lipoprotein; SD, standard deviation; SMD, standardized mean difference; T2DM, type 2 diabetes mellitus

Patients with concomitant CD demonstrated significantly increased risks of several diabetes-related complications. Albuminuria occurred in 7.5% of patients with T2DM + CD compared with 5.4% in matched controls (HR 1.30, 95% CI 1.15–1.46; *p* = 0.003). Neuropathy was also significantly more frequent in the CD cohort (17.3% vs. 9.8%; HR 1.73, 95% CI 1.59–1.88; *p* < 0.001). Similarly, diabetic ophthalmic complications occurred in 7.3% versus 3.9% of patients, respectively (HR 1.77, 95% CI 1.55–2.02; *p* < 0.001).

Psychiatric comorbidities were likewise more prevalent in the CD cohort. Depression occurred in 28.2% of patients with T2DM + CD versus 23.8% in controls (HR 1.14, 95% CI 1.07–1.21; *p* = 0.008), while anxiety occurred in 33.9% versus 29.6%, respectively (HR 1.12, 95% CI 1.06–1.18; *p* = 0.001).

Markers of poor glycemic control were also more common among patients with concomitant CD. Severe hyperglycemia with HbA1c ≥ 10% occurred in 12.5% of patients with T2DM + CD compared with 8.7% of matched controls (HR 1.39, 95% CI 1.27–1.53; *p* < 0.001), suggesting worse metabolic control despite matching for baseline HbA1c and diabetes-related therapies. When restricted to the first post-index occurrence of HbA1c ≥ 10%, severe hyperglycemia remained significantly more common in the CD cohort (6.5% vs. 5.1%; HR 1.16, 95% CI 1.01–1.32; *p* = 0.030), indicating that the association was not solely attributable to prevalent cases of poor glycemic control captured under the general definition (Table 2).

**Table 2:** Clinical outcomes after propensity score matching

Outcome	T2DM + CD (*n* = 8,760)	T2DM Without CD (*n* = 8,760)	HR (95% CI)	*p*-value
Albuminuria	660 (7.5%)	477 (5.4%)	1.30 (1.15–1.46)	0.003
Depression	2,467 (28.2%)	2,082 (23.8%)	1.14 (1.07–1.21)	0.008
Anxiety	2,973 (33.9%)	2,594 (29.6%)	1.12 (1.06–1.18)	0.001
HbA1c ≥ 10% *	1,094 (12.5%)	758 (8.7%)	1.39 (1.27–1.53)	< 0.001
HbA1c ≥ 10% (First Post-Index Occurrence) **	417 (6.5%)	417 (5.1%)	1.16 (1.01–1.32)	0.030
Ophthalmic complications	640 (7.3%)	343 (3.9%)	1.77 (1.55–2.02)	< 0.001
Hospitalization	3,181 (36.3%)	3,102 (35.4%)	0.97 (0.92–1.01)	0.110
Neuropathy	1,515 (17.3%)	861 (9.8%)	1.73 (1.59–1.88)	< 0.001
Nephropathy	1,385 (15.8%)	1,156 (13.2%)	1.13 (1.04–1.22)	< 0.001
Composite cardiovascular outcome	1,204 (13.7%)	1,087 (12.4%)	1.09 (1.01–1.17)	0.018

Abbreviations: CD, celiac disease; HbA1c, hemoglobin A1c; HR, hazard ratio; T2DM, type 2 diabetes mellitus. * HbA1c ≥10% was defined as HbA1c measurement ≥10% occurring after the index date, regardless of pre-index HbA1c ≥10% (no patients were excluded from this analysis). **First post-index occurrence of HbA1c ≥10% was defined as the first recorded HbA1c measurement ≥10% occurring after the index date, excluding patients with pre-index HbA1c ≥10%

## Discussion

In this large, multicenter, retrospective propensity score–matched cohort study, we found that concomitant celiac disease in patients with T2DM was associated with significantly higher risks of diabetic microvascular complications and greater psychiatric comorbidity. To our knowledge, this is the largest study to systematically evaluate the impact of concomitant CD on diabetes-related outcomes specifically in a T2DM population.

Our findings extend prior observations from T1DM cohorts to the T2DM population. Our study suggests that this pattern of increased microvascular risk is not confined to T1DM but extends to T2DM, suggesting that the pathophysiologic mechanisms linking CD to microvascular injury may operate independently of the autoimmune pathogenesis of T1DM.

Several mechanisms may explain the observed associations. CD is a systemic autoimmune disorder in which chronic intestinal inflammation and increased intestinal permeability can drive systemic immune activation and endothelial dysfunction [2, 15]. Additionally, CD-related malabsorption of micronutrients, including vitamins B12, D, E, copper, and folate, may contribute to neuropathy and other complications through both nutritional deficiency and impaired tissue repair [3, 16]. Peripheral neuropathy in CD has been attributed to nutritional deficiencies, chronic inflammation, and immune cross-reactivity, including anti-gliadin antibody binding to neuronal synapsin [4, 17]. In the context of T2DM, where hyperglycemia-driven microvascular injury is already present, these additional insults from CD may plausibly act synergistically to accelerate complication development; however, this proposed synergy remains speculative, as our study design did not directly test for interaction between CD-related mechanisms and hyperglycemia-driven injury, and should be evaluated in future mechanistic and prospective studies.

The significantly higher rate of severe hyperglycemia (HbA1c ≥ 10%) in the CD cohort despite matching for baseline HbA1c and diabetes therapies warrants particular attention. CD can impair nutrient absorption and complicate carbohydrate counting and mealtime consistency, potentially disrupting glycemic management [18]. Furthermore, gluten-free products often have a high glycemic index, which may paradoxically worsen glycemic control [18]. The association between CD, gluten-free diet, and the development of T2DM is inconsistent; some studies suggest that highly processed gluten-free diets may increase the risk of elevated glucose levels [19]. Gluten-free products are often formulated with refined starches such as rice, corn, tapioca, and potato, resulting in a higher glycemic index than their gluten-containing counterparts. In addition, commercially available GF pizza, bread, and pasta contain significantly more sugar, with studies reporting increases of up to 45% compared with conventional products, which may contribute to poorer glycemic control despite adherence to a gluten-free diet [3, 20]. The added burden of managing two chronic conditions may also reduce treatment adherence and self-management capacity, contributing to glycemic deterioration over time.

The increased prevalence of depression and anxiety in our CD cohort is consistent with a substantial body of literature demonstrating elevated psychiatric risk in CD. A large population-based study by Alkhayyat et al. found that patients with CD had significantly higher odds of depression (OR 1.92) and anxiety (OR 1.39) compared with those without CD [21]. A Danish nationwide cohort study similarly reported that CD was associated with increased risks of anxiety, depression, eating disorders, and stress, with the cumulative incidence of any neuropsychiatric disorder reaching 35.9% at 15 years of follow-up [22]. The mechanisms underlying this association remain incompletely understood but may involve disturbances in the gut–brain axis, chronic inflammation, nutrient deficiencies, and the psychosocial burden of dietary restriction [17]. In the context of T2DM, where depression and anxiety are already prevalent and independently associated with worse glycemic control and increased complication risk, the additive psychiatric burden of CD may further compound disease management challenges.

However, the observed associations between celiac disease and psychiatric outcomes must be interpreted with particular caution given the potential for unmeasured confounding. Propensity score methods adjust only for measured baseline covariates and make no claim to remove confounding due to unmeasured variables [23–25]. Several unmeasured factors may independently increase both the likelihood of celiac disease diagnosis and psychiatric comorbidity, including chronic gastrointestinal symptom burden, dietary restriction-related psychosocial stress, healthcare-seeking behavior, and the psychological impact of managing multiple chronic conditions [26, 27]. A large nationwide cohort study found that the risk of psychiatric disorders in celiac disease was highest in the first year after diagnosis, raising the possibility that surveillance may partly explain the association [27]. Furthermore, the modest effect sizes observed for depression (HR 1.14) and anxiety (HR 1.12) fall within a range where residual confounding alone could plausibly account for the entirety of the observed signal [28]. 

The composite cardiovascular outcome was modestly elevated in the CD cohort (HR 1.09, 95% CI 1.01–1.17). The relationship between CD and cardiovascular disease remains debated. A recent large propensity-matched analysis using the TriNetX Global Collaborative Network found that CD was not associated with excess cardiovascular risk after adjustment for comorbidities, and was in fact associated with lower risks of all-cause death and myocardial infarction [29]. However, a Swedish nationwide cohort study demonstrated a modest increase in ischemic heart disease risk among patients with CD (HR 1.19), and a UK Biobank phenome-wide association study reported elevated odds of atherosclerosis and heart failure in CD [30]. The modest signal for this composite cardiovascular outcome in our study may reflect the combined inflammatory and metabolic burden of coexisting CD and poorly controlled T2DM rather than CD alone as a risk factor and warrants further investigation in prospective studies.

Notably, hospitalization risk did not differ significantly between cohorts (HR 0.97), suggesting that the increased complication burden associated with CD in T2DM may not translate into greater acute healthcare utilization within the follow-up period studied. The relatively young age of the cohort and available follow-up duration may have limited the ability to detect differences in hospitalization.

It is important to note that the magnitude of several associations, including anxiety (HR 1.12), depression (HR 1.14), and nephropathy (HR 1.13), was modest, and these findings, together with the larger effects observed for neuropathy and ophthalmic complications, should be interpreted as hypothesis-generating associations rather than as validated tools for individual-level risk stratification or prediction.

This study has several limitations inherent to its retrospective, electronic health record–based design. First, the TriNetX platform relies on ICD-10-CM codes for disease identification, which may introduce misclassification bias. Although we enhanced diagnostic specificity for CD by requiring either markedly elevated tissue transglutaminase IgA levels or documented upper endoscopy in addition to the ICD-10 code, this algorithm does not confirm biopsy-proven CD in every patient, and some heterogeneity in case ascertainment may remain; patients with less clinically definitive or evolving CD presentations could have been included, potentially biasing effect estimates toward or away from the null. We also could not assess gluten-free diet adherence, which is a critical determinant of CD-related outcomes [31, 32]. In addition, because patients with a pre-index HbA1c ≥ 10% were not excluded from the general severe hyperglycemia analysis, this outcome is not strictly incident in the same sense as the diagnosis-code–based outcomes and should be interpreted as reflecting the burden of qualifying HbA1c values captured during follow-up. The first post-index-occurrence analysis, which did exclude patients with a pre-index HbA1c ≥ 10%, more closely approximates a strictly incident event. Second, despite propensity score matching, residual confounding from unmeasured variables—including CD duration, disease activity, dietary adherence, smoking status, and socioeconomic factors—cannot be excluded. Notably, several matching variables retained statistically significant imbalances after matching, although standardized mean differences were generally reduced. In particular, the CD cohort remained approximately 4 kg/m² lower in BMI than matched controls after matching (SMD 0.472), the largest residual imbalance among all matched covariates. Because the TriNetX platform’s built-in matching tools do not support multivariable regression adjustment beyond propensity score matching, we were unable to perform a sensitivity analysis directly adjusting for BMI and the other residually imbalanced covariates. This residual BMI imbalance should therefore be regarded as an important and unquantified potential source of confounding for outcomes with a substantial metabolic or adiposity component, including severe hyperglycemia and the composite cardiovascular outcome. Third, EHR-based data are subject to ascertainment bias, as patients with CD and T2DM may have more frequent healthcare encounters, leading to greater detection of complications [33]. Fourth, the TriNetX platform does not provide granular data on cause of death, and death data may be incomplete, potentially affecting time-to-event analyses [34]. Fifth, the observational nature of this study precludes causal inference, and the associations observed may reflect shared pathophysiologic pathways, surveillance bias, or unmeasured confounders rather than a direct causal effect of CD on diabetic complications. Finally, the study population was drawn from U.S. healthcare organizations, which may limit generalizability to other populations.

In this large propensity score–matched cohort, coexisting celiac disease was associated with a higher incidence of several clinically relevant outcomes among adults with T2DM. Although these findings support the hypothesis that CD may be associated with a distinct clinical profile in T2DM, the observational design precludes causal inference. Prospective studies incorporating detailed clinical phenotyping, disease duration, gluten-free diet adherence, and other potential confounders are needed to clarify the mechanisms underlying these associations.

## Supplementary information

Below is the link to the electronic supplementary material.

Supplementary Material 1

## Data Availability

The data that support the findings of this study were obtained from the TriNetX Research USA Minimal Date Shift network, a federated electronic health record database. Restrictions apply to the availability of these data, which were used under institutional license for the current study and are not publicly available. Aggregate de-identified data may be accessed through the TriNetX platform by authorized users and participating healthcare organizations.
