# Supplementary material for: Association of coexisting celiac disease with macrovascular and microvascular complications in adults with type 2 diabetes mellitus: a retrospective propensity score–matched cohort study
Source: Endocrine. 2026 Sep 28;91(1):314. doi: 10.1007/s12020-026-04769-3 (PMC13619779; doi:10.1007/s12020-026-04769-3)
Supplement: Supplementary file 1 [file 12020_2026_4769_MOESM1_ESM.docx]

**Supplementary Materials**

**Supplementary Material 1: Inclusion and Exclusion Criteria of Cohorts**

Two cohorts were defined within the TriNetX Research USA Minimal Date Shift network (56 healthcare organizations). Cohort 1 required co-occurrence of T2DM (E11) and celiac disease (K90.0) with serologic or endoscopic confirmation. Cohort 2 included patients with T2DM only, excluding celiac disease and type 1 diabetes mellitus.

| **Domain** | **Cohort 1: T2DM + Celiac Disease** | **Cohort 2: T2DM Without Celiac Disease (Controls)** |
| --- | --- | --- |
| **Study Period** | All dates available (no date restriction applied) | All dates available (no date restriction applied) |
| **Inclusion Criteria** | All of the following:   - Type 2 diabetes mellitus (ICD-10-CM: E11) - Celiac disease (ICD-10-CM: K90.0)   AND at least one of the following confirmatory criteria:   - Elevated tissue transglutaminase IgA ≥200 arb'U/L (LOINC: 31017-7) - Endoscopy of the esophagus/upper GI (CPT: 1007241) | All of the following:   - Type 2 diabetes mellitus (ICD-10-CM: E11)   AND none of the following:   - Celiac disease (ICD-10-CM: K90.0) - Type 1 diabetes mellitus (ICD-10-CM: E10) |
| **Exclusion Criteria** | - Did not meet both T2DM (E11) and celiac disease (K90.0) criteria - Lacked confirmatory serology (tTG-IgA ≥200) or endoscopy (CPT: 1007241) - Index event occurred more than 20 years prior to analysis (0 patients excluded) | - Any diagnosis of celiac disease (K90.0) - Any diagnosis of type 1 diabetes mellitus (E10) - Index event occurred more than 20 years prior to analysis (0 patients excluded) |
| **Index Event** | First co-occurrence of T2DM diagnosis (E11), celiac disease (K90.0), and confirmatory serology or endoscopy | First recorded diagnosis of type 2 diabetes mellitus (E11) |
| **Outcome Time Window** | 30 days after index event onward (no end date; all outcomes included thereafter) | 30 days after index event onward (no end date; all outcomes included thereafter) |

*Abbreviations: CPT = Current Procedural Terminology; ICD-10-CM = International Classification of Diseases, 10th Revision, Clinical Modification; LOINC = Logical Observation Identifiers Names and Codes; PSM = Propensity Score Matching; T2DM = type 2 diabetes mellitus; tTG-IgA = tissue transglutaminase IgA antibody. All outcomes were assessed from 30 days after the index event, with no upper time limit.*

**Supplementary Material 2: Covariates Included in the Propensity Score Model**

Propensity score matching (1:1 nearest-neighbor matching) was performed on 14 baseline characteristics across four domains. After matching, 8,760 patients were retained in each cohort from an initial pool of 8,770 (Cohort 1) and 6,294,516 (Cohort 2) patients.

| **Domain** | **Variables Matched** |
| --- | --- |
| **Demographics** | Current age (continuous); sex (male).  Race/ethnicity categories:  White; Black or African American; Asian; Other Race; Hispanic or Latino; Not Hispanic or Latino. |
| **Diagnoses** | Disorders of lipoprotein metabolism and other lipidemias (ICD-10-CM: E78);  Essential (primary) hypertension (ICD-10-CM: I10). |
| **Medications** | Antilipemic agents (ATC: CV350);  Insulins and analogues (ATC: A10A);  Metformin (RXNORM: 6809). |
| **Laboratory Values** | Hemoglobin A1c / Hemoglobin total in blood (LOINC: 9037) — continuous;  Cholesterol in LDL [Mass/volume] in serum or plasma (LOINC: 9002) — continuous;  Body mass index — BMI (LOINC: 9083) — continuous. |

*Abbreviations: ATC = Anatomical Therapeutic Chemical classification; BMI = body mass index; HbA1c = hemoglobin A1c; LDL = low-density lipoprotein; LOINC = Logical Observation Identifiers Names and Codes; PSM = Propensity Score Matching; RXNORM = National Library of Medicine RxNorm; SMD = standardized mean difference; T2DM = type 2 diabetes mellitus.*

**Supplementary Material 3: Outcomes and Corresponding ICD-10 Codes**

All outcomes were assessed from 30 days after the index event with no upper time limit, using both risk analysis and Kaplan-Meier survival analysis. Patients with outcomes prior to the time window were included in all analysis.

| **Outcome** | **ICD-10-CM Code(s) and Description** | **Analysis Settings** |
| --- | --- | --- |
| **Albuminuria** | **ICD-10-CM: R80** — Proteinuria | *Risk analysis and Kaplan-Meier survival analysis: including patients with outcome prior to the time window* |
| **Depression** | **ICD-10-CM: F32** — Depressive episode | *Risk analysis and Kaplan-Meier survival analysis: including patients with outcome prior to the time window* |
| **Anxiety** | **ICD-10-CM: F41** — Other anxiety disorders | *Risk analysis and Kaplan-Meier survival analysis: including patients with outcome prior to the time window* |
| **Poor glycemic control (HbA1c ≥10%)** | **ICD-10-CM: LOINC: 9037** — Hemoglobin A1c/Hemoglobin.total in Blood (most recent value ≥10.00%) | *Risk analysis and Kaplan-Meier survival analysis: including patients with outcome prior to the time window* |
| **Diabetic eye complications** | **ICD-10-CM: E11.3** — Type 2 diabetes mellitus with ophthalmic complications | *Risk analysis and Kaplan-Meier survival analysis: including patients with outcome prior to the time window* |
| **Hospitalization** | **ICD-10-CM: CPT/Visit: IMP** — Inpatient encounter (HL7 visit type: IMP) | *Risk analysis and Kaplan-Meier survival analysis: including patients with outcome prior to the time window* |
| **Diabetic neuropathy** | **ICD-10-CM: E11.4** — Type 2 diabetes mellitus with neurological complications | *Risk analysis and Kaplan-Meier survival analysis: including patients with outcome prior to the time window* |
| **Diabetic nephropathy** | **ICD-10-CM: E11.2** — Type 2 diabetes mellitus with kidney complications | *Risk analysis and Kaplan-Meier survival analysis: including patients with outcome prior to the time window* |
| **Major Adverse Cardiovascular Events (MACE)** | **ICD-10-CM: I21** — Acute myocardial infarction  **ICD-10-CM: I22** — Subsequent STEMI and NSTEMI myocardial infarction  **ICD-10-CM: I46** — Cardiac arrest  **ICD-10-CM: I50** — Heart failure  **ICD-10-CM: I61** — Nontraumatic intracerebral hemorrhage  **ICD-10-CM: I62** — Other nontraumatic intracranial hemorrhage  **ICD-10-CM: I63** — Cerebral infarction  **ICD-10-CM: I65** — Occlusion and stenosis of precerebral arteries, no infarction  **ICD-10-CM: I66** — Occlusion and stenosis of cerebral arteries, no infarction  **ICD-10-CM: I49.0** — Ventricular fibrillation and flutter  **ICD-10-CM: I49.3** — Ventricular premature depolarization  **ICD-10-CM: I67.8** — Other specified cerebrovascular diseases  **ICD-10-CM: I67.9** — Cerebrovascular disease, unspecified | *Risk analysis and Kaplan-Meier survival analysis: including patients with outcome prior to the time window* |

*Abbreviations: ICD-10-CM = International Classification of Diseases, 10th Revision, Clinical Modification; LOINC = Logical Observation Identifiers Names and Codes; MACE = major adverse cardiovascular events; NSTEMI = Non-ST-Elevation Myocardial Infarction; STEMI = ST-Elevation Myocardial Infarction; T2DM = type 2 diabetes mellitus. All outcomes were assessed from 30 days after the index event, with no upper time limit.*

**Supplementary Material 2: Covariates Included in the Propensity Score Model**

Propensity score matching (1:1 nearest-neighbor matching) was performed on 14 baseline characteristics across four domains. After matching, 8,760 patients were retained in each cohort from an initial pool of 8,770 (Cohort 1) and 6,294,516 (Cohort 2) patients.

| **Domain** | **Variables Matched** |
| --- | --- |
| **Demographics** | Current age (continuous); sex (male).  Race/ethnicity categories:  White; Black or African American; Asian; Other Race; Hispanic or Latino; Not Hispanic or Latino. |
| **Diagnoses** | Disorders of lipoprotein metabolism and other lipidemias (ICD-10-CM: E78);  Essential (primary) hypertension (ICD-10-CM: I10). |
| **Medications** | Antilipemic agents (ATC: CV350);  Insulins and analogues (ATC: A10A);  Metformin (RXNORM: 6809). |
| **Laboratory Values** | Hemoglobin A1c / Hemoglobin total in blood (LOINC: 9037) — continuous;  Cholesterol in LDL [Mass/volume] in serum or plasma (LOINC: 9002) — continuous;  Body mass index — BMI (LOINC: 9083) — continuous. |

*Abbreviations: ATC = Anatomical Therapeutic Chemical classification; BMI = body mass index; HbA1c = hemoglobin A1c; LDL = low-density lipoprotein; LOINC = Logical Observation Identifiers Names and Codes; PSM = Propensity Score Matching; RXNORM = National Library of Medicine RxNorm; SMD = standardized mean difference; T2DM = type 2 diabetes mellitus.*

**Supplementary Material 3: Outcomes and Corresponding ICD-10 Codes**

All outcomes were assessed from 30 days after the index event with no upper time limit, using both risk analysis and Kaplan-Meier survival analysis. Patients with outcomes prior to the time window were included in all analyses.

| **Outcome** | **ICD-10-CM Code(s) and Description** | **Analysis Settings** |
| --- | --- | --- |
| **Albuminuria** | **ICD-10-CM: R80** — Proteinuria | *Risk analysis and Kaplan-Meier survival analysis: including patients with outcome prior to the time window* |
| **Depression** | **ICD-10-CM: F32** — Depressive episode | *Risk analysis and Kaplan-Meier survival analysis: including patients with outcome prior to the time window* |
| **Anxiety** | **ICD-10-CM: F41** — Other anxiety disorders | *Risk analysis and Kaplan-Meier survival analysis: including patients with outcome prior to the time window* |
| **Poor glycemic control (HbA1c ≥10%)** | **ICD-10-CM: LOINC: 9037** — Hemoglobin A1c/Hemoglobin.total in Blood (most recent value ≥10.00%) | *Risk analysis and Kaplan-Meier survival analysis: including patients with outcome prior to the time window* |
| **Diabetic eye complications** | **ICD-10-CM: E11.3** — Type 2 diabetes mellitus with ophthalmic complications | *Risk analysis and Kaplan-Meier survival analysis: including patients with outcome prior to the time window* |
| **Hospitalization** | **ICD-10-CM: CPT/Visit: IMP** — Inpatient encounter (HL7 visit type: IMP) | *Risk analysis and Kaplan-Meier survival analysis: including patients with outcome prior to the time window* |
| **Diabetic neuropathy** | **ICD-10-CM: E11.4** — Type 2 diabetes mellitus with neurological complications | *Risk analysis and Kaplan-Meier survival analysis: including patients with outcome prior to the time window* |
| **Diabetic nephropathy** | **ICD-10-CM: E11.2** — Type 2 diabetes mellitus with kidney complications | *Risk analysis and Kaplan-Meier survival analysis: including patients with outcome prior to the time window* |
| **Major Adverse Cardiovascular Events (MACE)** | **ICD-10-CM: I21** — Acute myocardial infarction  **ICD-10-CM: I22** — Subsequent STEMI and NSTEMI myocardial infarction  **ICD-10-CM: I46** — Cardiac arrest  **ICD-10-CM: I50** — Heart failure  **ICD-10-CM: I61** — Nontraumatic intracerebral hemorrhage  **ICD-10-CM: I62** — Other nontraumatic intracranial hemorrhage  **ICD-10-CM: I63** — Cerebral infarction  **ICD-10-CM: I65** — Occlusion and stenosis of precerebral arteries, no infarction  **ICD-10-CM: I66** — Occlusion and stenosis of cerebral arteries, no infarction  **ICD-10-CM: I49.0** — Ventricular fibrillation and flutter  **ICD-10-CM: I49.3** — Ventricular premature depolarization  **ICD-10-CM: I67.8** — Other specified cerebrovascular diseases  **ICD-10-CM: I67.9** — Cerebrovascular disease, unspecified | *Risk analysis and Kaplan-Meier survival analysis: including patients with outcome prior to the time window* |

*Abbreviations: ICD-10-CM = International Classification of Diseases, 10th Revision, Clinical Modification; LOINC = Logical Observation Identifiers Names and Codes; MACE = major adverse cardiovascular events; NSTEMI = Non-ST-Elevation Myocardial Infarction; STEMI = ST-Elevation Myocardial Infarction; T2DM = type 2 diabetes mellitus. All outcomes were assessed from 30 days after the index event, with no upper time limit.*
